# The food security of residents and refugees of Ukraine after the Russian invasion

**DOI:** 10.1038/s41598-025-99285-1

**Published:** 2025-05-09

**Authors:** Olha Nimko, Leonie Hodel, Adelina Chandra, Rachael Garrett

**Affiliations:** 1 Environmental Policy Lab, Department of Environmental Systems Science, ETH Zurich, Zürich, Switzerland; 2 Department of Geography and Conservation Research Institute, University of Cambridge, Cambridge, UK

**Keywords:** War, Nutrition, Migration, Displacement, Environmental social sciences, Environmental economics, Sustainability

## Abstract

In this paper, survey evidence is used to examine the food security impacts of the 2022 Russian invasion of Ukraine. We focus on both residents of Ukraine and those fleeing to one of the wealthiest countries in Europe, Switzerland. Our questionnaire was sent to both Ukrainian residents and migrants to Switzerland between October 2022 and February 2023. 80% of respondents (n = 1267) indicated that they faced some form of food insecurity, most commonly an inability to eat balanced meals and/or running out of food without being able to buy more. Yet the incidence of more severe forms of food insecurity, including cutting and skipping meals for multiple months, eating less than needed, and feeling hungry without eating, affected more than 20% of the surveyed populations in both countries. Food insecurity for both residents of Ukraine and refugees in Switzerland is significantly more severe for households who perceive themselves to have below average income. In Ukraine higher food insecurity is identified in the conflict’s frontline regions and among larger households with less domestic food production. In Switzerland, women report lower access to many coping mechanisms (producing, trading, or borrowing food). This study underscores the urgent need for improved food programs in conflict zones as well as countries that host refugees, particularly where domestic food prices are very high and language barriers reduce income opportunities.

## Introduction

Conflict is one of the largest drivers of food insecurity globally with more than half of the world’s undernourished living in areas affected by conflict^1^. Military activities and violence directly threaten food production and access, but also lead to the displacement of populations to places where they lack employment, social networks, or direct means of food production^2^. Indeed, conflict is the second largest driver of forced migration globally, accounting for 46% of the world’s 110 million displaced people^3^. Due to a lack of financial, physical, and social resources, refugees fleeing conflict are significantly more likely to face challenges accessing sufficient safe and nutritious food than other residents of host countries^4^.

In February 2022, Russia’s invasion of Ukraine became a central driver of refugee movements in Europe, leading to the displacement of more than 3 million people within Ukraine and 6 million people abroad^5^. Besides directly impacting the food security of people forced to find new homes, the conflict has significantly disrupted the production and supply of food in Ukraine^6^. As one woman reported in a 2024 OECD report, “*It was very scary, the children were crying, the food was running out…My husband stayed to protect our native land, and I decided to save [our] children from the war.*”^7^ Nearly two years after the Russian invasion, farmland at the front line is unusable, numerous livestock have been killed, many stores are not operating, and Ukrainians have to contend with Russian soldiers looting stores and homes for food and water^8^. Martial law has also impacted the agricultural sector through a lack of labor and restrictions on travel within the country and abroad^9^.

Here we investigated how the Russian invasion of Ukraine, Europe’s largest conflict in the past decade, affected food security (defined as access to sufficient, safe and nutritious food to meet dietary needs and food preferences) among Ukrainians who stayed, as well as refugees. In 2022 the UN World Food Program (WFP) had estimated that one-fifth of the population residing in Ukraine is already getting less food because of the war, and adults are skipping meals so that children can eat^10^. In 2024 another study was published indicating that one-third of the population had experienced some form of food insecurity, based on surveys from early 2022^2^. We provide an update to this estimate within Ukraine as well as insights into the situation of Ukrainians who relocated to Switzerland, which received over 60,000 refugees from Ukraine as of the end of 2023^11^, constituting more than 30% of the total number of refugees hosted by Switzerland in 2022 and 2023^5^.

We analyze the results of a food security survey sent to all regions of Ukraine (UA) and Switzerland (CH) between October 2022 to February 2023. The responses of the survey (n = 1,160 UA, n = 107 CH) were used to create a food security index that measures the level of food insecurity (the higher the score, the higher the food insecurity). We then econometrically examine how household characteristics are linked to food insecurity outcomes. In particular we are seeking to understand which types of households are more likely to suffer from greater food insecurity. Given our focus on consuming households we focus on factors that influence the ability to pay for or physically access food relative to household needs. These factors include income, household size, education and awareness, gender, and restrictions on movement which can affect travel to markets (only relevant in the active conflict zone—Ukraine). We also look at how consumption of home- or locally-produced, bartered, or gifted food and access to food aid are correlated with food insecurity, since these mechanisms can offset reductions in income and movement, improving overall food security outcomes^12^.

While theories about links between conflict and food insecurity largely focus on the countries where the conflict takes place, we examine whether they persist among refugees. Such data are rare in the literature on food security, particularly for refugees within Europe^13^, but income, household size, and access to food aid and food production have been found to be relevant factors for refugee food security in Lebanon^14,15^, South Africa^16^, and the United States^17^. By simultaneously conducting surveys across Ukraine and Switzerland we have a unique opportunity to compare how refugees from the Ukraine-Russia war to Switzerland, one of the richest countries in Europe^18^, have fared compared to people who have remained in the conflict-affected region. Previous studies have shown that refugees to wealthy countries often experience low incomes relative to the resident population and struggle with grocery shopping and language barriers and have a smaller support network. All of these factors can reduce food security^19,20^. Consequently, food insecurity may be equally bad or worse for refugees than those that have remained in conflict-affected regions, depending on the context.

## Methods

### Food measure

We use an experience-based food [in]security scale, the US Household Food Security Survey Module, rather than a consumption-based measure^21^. This survey approach is similar to the Household Food Insecurity Access Scale of the UN WFP and the FAO Food Insecurity Experience Scale, among others, and has been found to be a valid measure of food insecurity in many countries worldwide^22^. The experiential measure recognizes that food insecurity starts with anxiety and worry, before affecting food purchases and leftovers, then the quantity, quality, and diversity of food consumed^23,24^.

Our survey approach combined questions from the U.S. Household Food Security Survey Module with additional questions from the UN Refugee Agency’s (UNHCR) standardized expanded nutrition survey for refugee populations^25^. Modifications were made to focus on the period since the Invasion of Ukraine on February 24, 2022. Following the methodology of the U.S. Household Food Security Survey, an index was created from six core questions in the survey. The first food security question [HH1] is a preliminary screening question “Which of these statements best *describes the food eaten in your household* since the Invasion of Ukraine, February 24, 2022?” [1] Enough of the kinds of food I/we want to eat, [2] Enough but not always the kinds of food I/we want, [3] Sometimes not enough to eat, or [4] Often not enough to eat. If the answer is 1, the household is given a score of 0. If the response is anything other than 1, they proceed with the rest of the questions that create the food security score.

### Data collection

The study was performed in accordance with relevant human subject guidelines and received approval from the ETH Zürich Ethics Commission. It was translated into Ukrainian, which is spoken by 99 + % of the population^26^ and all participants provided informed consent before taking the survey. Beside the food security module questions, and questions about food aid and other coping mechanisms, each participant was asked to provide information on age, level of education, perceived income in 2021 relative to others, number of people in the household, gender, and nationality (Table S1-S2). While we could have collected more data on household characteristics determining food security, we purposefully kept the survey concise (see SM1 for full survey) so that respondents could conveniently answer it without taking up too much of their time, given their existing stress.

The intended audience for this survey consisted of one representative per household who met the criteria of being 18 years or older and belonged to one of the following categories: Ukrainian refugees residing in Switzerland, or Ukrainian nationals who were either unable or unwilling to evacuate from Ukraine. The scope of this assessment encompasses the entirety of the territories of Switzerland and Ukraine, except for areas that were directly under occupation and control by the Russian army. As a result, there is no data on Crimea and Luhansk oblasts.

Data collection in both countries occurred from October 27, 2022 to February 5, 2023. In Ukraine the survey was sent to a panel of around 30,000 active internet-using households compiled by IMData. The household panel maintained by IMData is representative of the Ukrainian population with internet access and is stratified according to the socio-demographic structure of internet users in the country (i.e., by age, gender, and city/village), who comprise 80% of the population^27^. A total of 1364 respondents completed the survey online using the open-source KoboToolbox software (kobotoolbox.org), leading to a response rate of 4%. This rate is somewhat lower than the normal range for surveys distributed by email (5–10%), but relatively high considering the wartime situation and conscription of much of the adult male population. Comparing the descriptive statistics of our sample to available background demographic characteristics, we find our sample to be representative across many categories (see Table S2). 84 respondents reported living in the occupied territories of Ukraine and a further 13 contained incomplete responses. These households were removed from the sample leaving 1267 total responses across the two countries.

In Switzerland, the survey was distributed through Telegram channels and Facebook groups for Ukrainian refugees. We obtained responses across 23 of the 26 cantons, reflecting a strong reach into the refugee population within the country. Our sample also aligns well with the demographic characteristics of the refugee population as measured by UNHCR surveys during the same period (Table S2). We have slightly oversampled highly educated people, so our sample may be capturing people that may be somewhat better off than the average refugee population. Nevertheless, the number of people reporting self-perception of lower-than-average income aligns with the percentage of people noting income deprivation in the UNHCR survey (Table S2).

### Analysis

Per the standard methodology of the US Household Food Security Survey the six food security questions were used to generate a single food security index score. The full set of questions can be found in the Supplementary Material and the responses are visible in Fig. 2. The index includes responses to HH3, HH4, AD1, AD2, and AD3. Responses of “often” or “sometimes” on questions HH3 and HH4, and “yes” on AD1, AD2, and AD3 are coded as affirmative (yes). Responses of “almost every month” and “some months but not every month” on question AD1a are coded as affirmative (yes). Each yes equals a score of 1 and the sum is the household’s raw score on the scale. If the household’s total raw score is 0–1, they are characterized as having high or marginal food security. A raw score of 2–4 indicates low food security. A raw score of 5–6 indicates very low food security. Additional questions asked in the survey can be found in the Supplementary Material.

The respondents surveyed represent a diverse range of nationalities, including Ukrainian, Russian, Polish, Tatar, Jewish, Lithuanian, Belarusian, Chuvash, and Turkish. The largest proportion of respondents are women and people in the 26–49 age category (Table S2) largely due to mandatory military service for men in Ukraine. Furthermore, there were notable differences in income distribution, with a higher proportion of respondents reporting below average or average income in Ukraine and a more favorable income distribution in Switzerland. Additionally, the share of respondents with higher education was higher in Switzerland, possibly influenced by the presence of skilled refugees and emigration (Table S2).

To assess the demand side drivers of food insecurity we regress income, location in frontline regions (Ukraine only), access to food aid, home food production, and household size on the food security index using cross-sectional models with and without regional or canton fixed effects:

$$\begin{gathered} {\text{Food Insecurity}}_{{\text{i}}} = \alpha + \beta_{{1}} {\text{Income}}_{{\text{i}}} + \beta_{{2}} {\text{Higher Education}}_{{{\text{i}} }} \hfill \\ + \beta_{{3}} {\text{Food Aid}}_{{\text{i}}} + \beta_{{4}} {\text{Self Production}}_{{\text{i}}} \hfill \\ + \beta_{{5}} {\text{Household Size}}_{{\text{i}}} + \beta_{{6}} {\text{Gender}}_{{\text{i}}} + \beta_{{7}} {\text{Frontline}}_{{\text{i}}} + \delta_{{\text{i}}} + \varepsilon_{{\text{i}}} \hfill \\ \end{gathered}$$

where α is the intercept, β_1-7_ are the coefficients for household determinants of food insecurity, δ_i_ represents the spatial fixed effect and ε_i_ is the error term. We also used Pearson correlation coefficients to examine the relationship between the food insecurity index and the background conditions (Figure S1).

A limitation for the correlational approaches is the low sample size for the Swiss data, which reduces the power for detecting significant relationships between food security and background conditions. The implication of limited power is less certainty that there is no effect when a variable has no statistical significance in the regression.

## Results

**Fig. 1:**
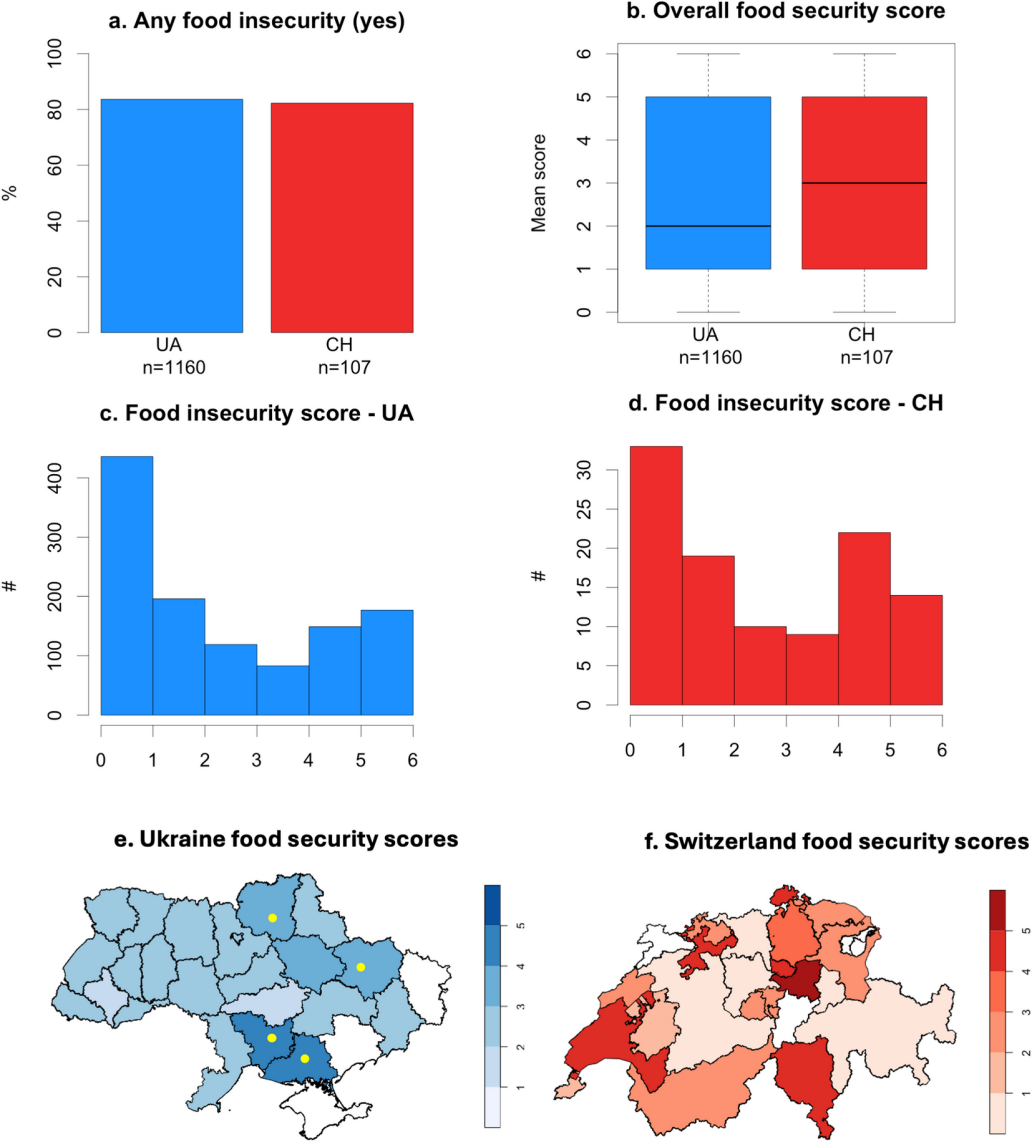
Overall food security outcomes for residents of Ukraine (UA) and Ukrainian refugees in Switzerland (CH). Panel (**a**) indicates the percentage in each region who reported any food insecurity. In panel (**b**), the boxplots show the mean and standard deviation across individual respondents (1 = high or marginal food security, 2–4 low food security, 5–6 very low food security), whilst panels (**c**) and (**d**) show these data as histograms for Ukraine and Switzerland, respectively. Panel (**e**) shows the mean score food security score by Ukrainian regions with frontline areas indicated by a yellow circle and panel (**f**) by Swiss canton with the scale shown on the right. Areas in white had no respondents.

We found a high degree of similarity in food insecurity scores across Ukrainian (UA) residents and refugees to Switzerland (CH) (Fig. 1a–d). Overall, 83.6% of the Ukrainian refugee sample population in Switzerland and 82.2% of those in the sample who remained in Ukraine had experienced some form of food insecurity after the invasion (Fig. 1a). However, the spread of food security scores across regions/cantons was significantly higher in Switzerland than Ukraine (min/max score UA = 1.97/4.33, CH = 0.43/5.4, Fig. 1e,f). Most respondents were women (UA = 68.1%, CH = 88.8%), likely due to required military service for me and restrictions on their travel.

**Fig. 2:**
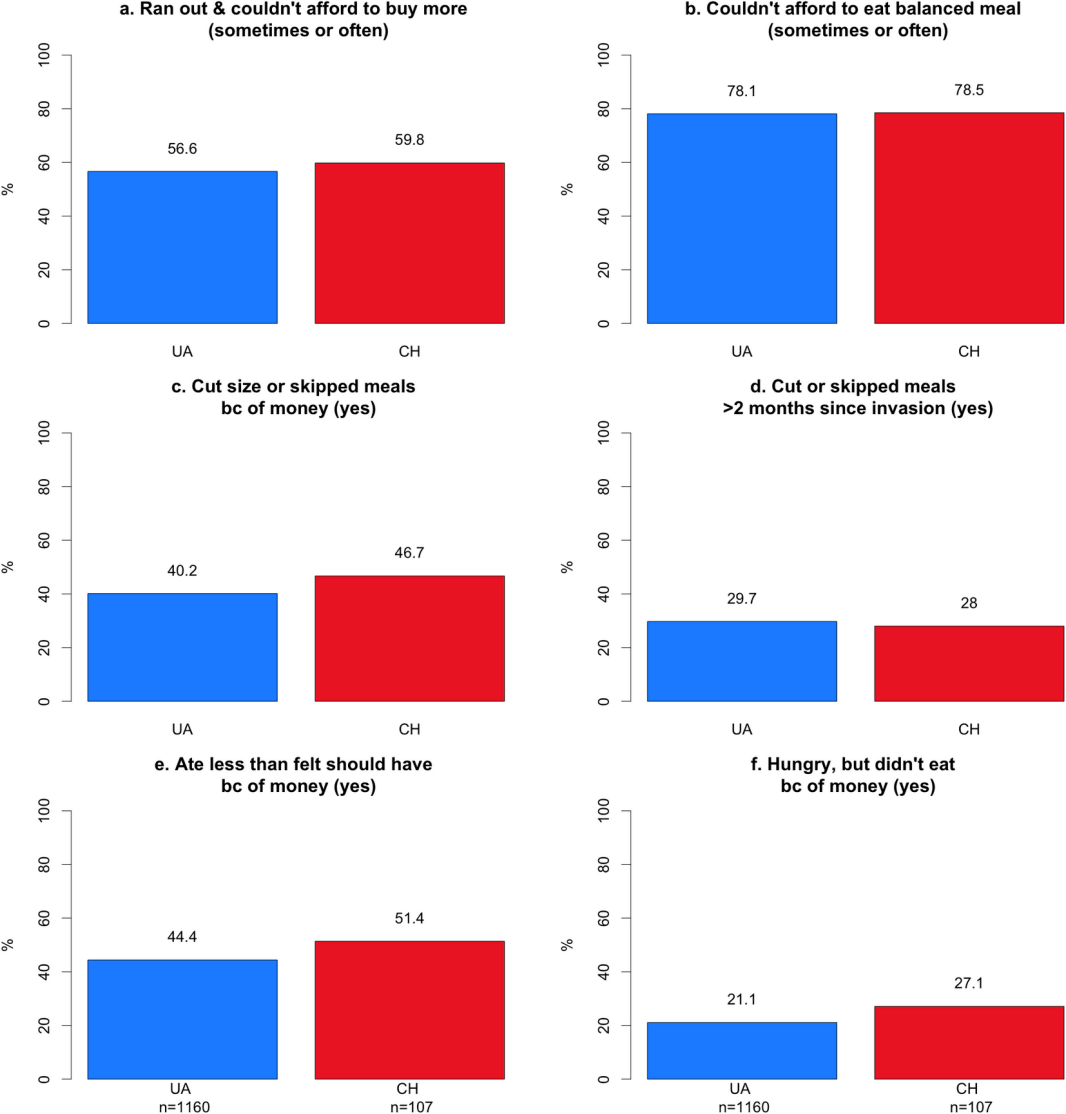
Responses to six questions underlying the food security index for residents of Ukraine (UA) and Ukrainian refugees in Switzerland (CH).

The most reported food security challenge in both regions was the inability to afford to eat a balanced meal (UA = 78.1%, CH = 78.5%; Fig. 2b), followed by running out of food and not being able to buy more (UA = 56.6%, CH = 59.8%; Fig. 2a). The need to eat less, either by cutting meal sizes, skipping meals, or eating less than one felt one should, was reported by 40–45% of the populations surveyed in each region (Fig. 2c,d). More severe impacts were less common; the incidence of hunger due to a lack of affordability was 21.1% in UA and 27.1% in CH. Weight loss occurred among 21.1% in UA and 27.1% in CH. The inability to eat for an entire day was rarer, 11.0% in UA and 8.4% in CH.

**Fig. 3:**
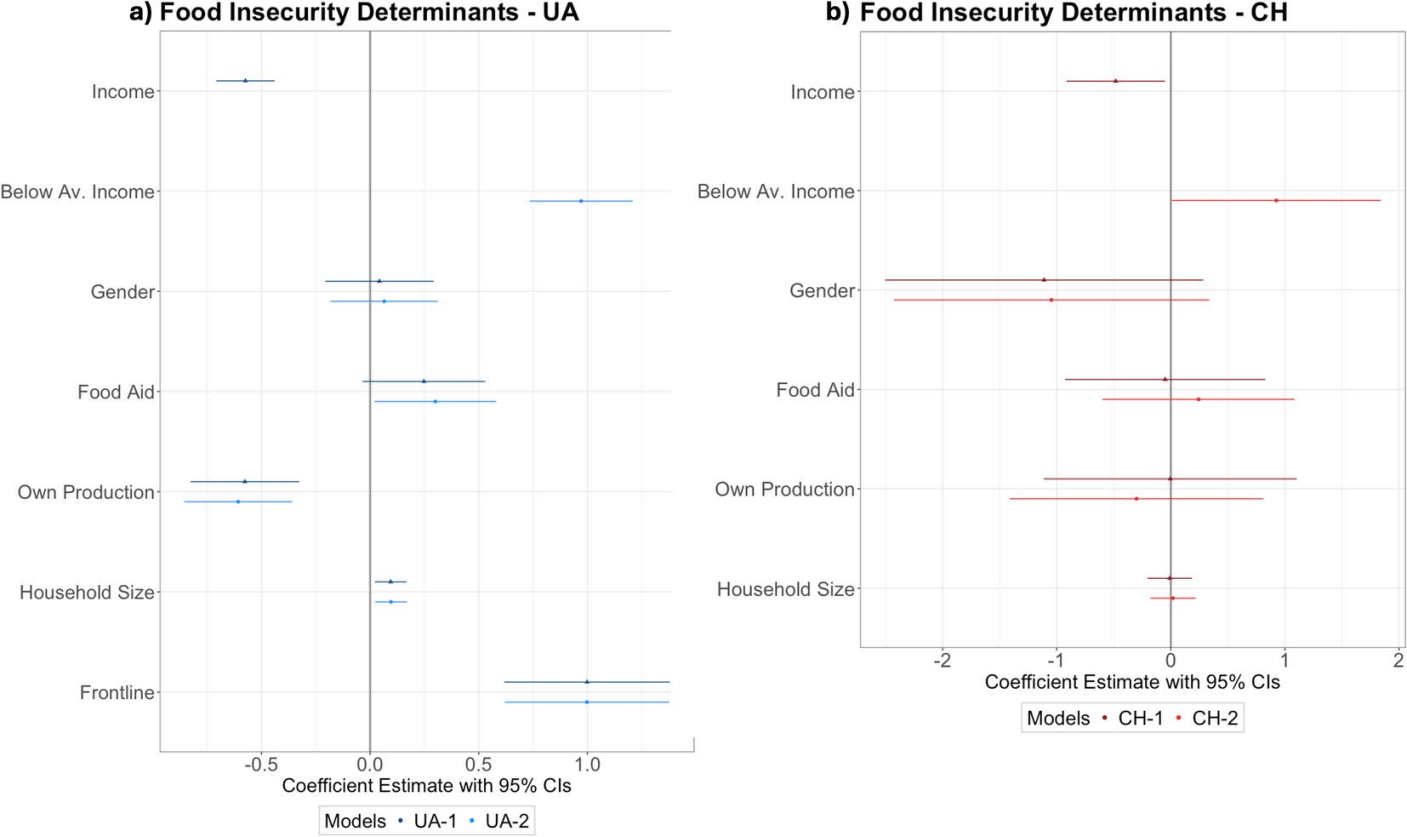
Coefficient plots with a 95% confidence interval for the demand side predictors of the food insecurity index in UA (**a**) and CH (**b**). Higher coefficients indicate a positive relationship with food insecurity. Coefficients overlapping with zero are not significant. Gender indicates female headed households. Food Aid, Own Production, and Frontline are all yes/no. Income and Household size are continuous variables.

As expected, households with lower perceived income relative to their peers as of 2021 reported significantly higher food insecurity in both regions (Fig. 3, Table S3). Perceived income group was negatively correlated with more severe forms of food insecurity (i.e., hunger, eating less, skipping meals), as well as a general lack of food affordability, particularly in Switzerland. While the percentage of Ukrainian residents with average or below average incomes reporting any type of food insecurity was only 9% higher than households with above average incomes in Ukraine (84% versus 75%), in Switzerland this difference between poorer and wealthier households in the prevalence of any food insecurity rose to 38% (88% versus 50%). Food insecurity was significantly higher in larger households in Ukraine, and higher in female headed refugee households in Switzerland, but with a large variation (Fig. 3, Table 1). This could be related to differences in economic opportunities, as women respondents in Switzerland reported lower income than male respondents.

**Table 1:** Ukraine regression results for the food insecurity index as the dependent variable.

Predictors	Model 1b—UA	Model 2b—UA
	Estimates (SE)	Estimates (CI)
Perceived relative income group (1 = lowest, 5 = highest)	− 0.57*** (0.24)	− 0.57*** (0.07)
Advanced education (1 = yes)	− 0.27*** (0.07)	− 0.24† (0.12)
Frontline (1 = yes)	1.00*** (0.12)	0.90† (0.47)
Food aid (1 = yes)	0.25*** (0.14)	0.23 (0.15)
Own production (1 = yes)	− 0.58*** (0.13)	− 0.59*** (0.13)
Household size	0.10*** (0.04)	0.10*** (0.04)
Gender (1 = female)	0.04*** (0.13)	0.00 (0.13)
FE	No	Yes
Observations	1120	1120
Adjusted R^2^	0.12	0.13

Main models found in main text. Model 2b includes FE are at the region/canton level. †p < 0.1, *p < 0.05, **p < 0.01, ***p < 0.001.

Within Ukraine, the frontline regions (Chernihiv, Mykolaiv, Kherson, and Donetsk oblasts) reported significantly higher food insecurity (Fig. 1c, Fig. 3, Table S1). This aligns with theory about the impacts of reduced mobility on food insecurity, as frontline regions in Ukraine have seen significantly reduced mobility due to damage to roadways and other transport infrastructure^28^. Within Switzerland, food insecurity was highest in the smaller cantons like Schaffhausen, Schwyz, Vaud, Solothurn, Zug, and Ticino (Fig. 1d, Table S1). The reasons for certain cantons having lower food insecurity rates are less clear since they are not explained by overall levels of asylum seekers in the canton or differences in income and employment across cantons (Table S4).

As for coping strategies, 48% of respondents in Ukraine and 33% in Switzerland managed their food insecurity through producing their own food or trading, borrowing, or being gifted food from others. In Ukraine the production of food at home was a significant factor in reducing reported household food insecurity (Fig. 3, Table 1). Within our sample 59% of the respondents in Switzerland had received food aid support, as compared to only 22% in Ukraine and this food aid access was inversely correlated with income in Switzerland (Pearson’s R = − 0.28, p = 0.007). Despite its higher prevalence, food aid in Switzerland had no significant correlation with food insecurity levels, while the relationship in Ukraine, where many fewer people received such support, was weakly positive (Fig. 3, Table 2).

**Table 2:** Switzerland regression results for the food insecurity index as the dependent variable.

Predictors	Model 1—CH	Model 2—CH
	Estimates (CI)	Estimates (CI)
Perceived relative income group (1 = lowest, 5 = highest)	− 0.48* (0.22)	− 0.43† (0.24)
Advanced education (1 = yes)	− 0.38 (0.48)	0.30 (0.53)
Food aid (1 = yes)	− 0.05 (0.45)	− 0.15 (0.53)
Own production (1 = yes)	− 0.01 (0.57)	0.04 (0.66)
Household size	− 0.01 (0.10)	0.06 (0.11)
Gender (1 = female)	− 1.11 (0.71)	− 0.78 (0.77)
Observations	1120	1120
Adjusted R^2^	0.117	0.123

## Discussion

Our study, conducted from October 2022 onwards, finds similar levels of food insecurity among Ukrainians that remained in the country as Rudolfsen et al.^2^, who surveyed the population starting in March–April 2022 using a different experienced-based food measure^29^. 40% of the respondents in our study reported “cutting or skipping meals since the invasion because of money” and 44% reported they “ate less than they felt they should have because of money”, whilst Rudolfsen et al.^2^ find that 36% of respondents experienced at least 1 day without food in the last seven days, and 45% experienced at least 1 day with limited meals. Together these results provide greater certainty that nearly half the resident Ukrainian non-military population has suffered from reduced food consumption.

The regression results confirm our expectation that families who perceive themselves to have below average income experience more food insecurity in both Ukraine and Switzerland. Indeed, in Switzerland, perceived income was the only household characteristic that had a statistically significant relationship with the food insecurity measure. This result is consistent with prior studies showing that perceived income is an important determinant of household behavior and resulting household health outcomes^30^. Though conceptually different than objective income, perceived income status can be a better measure than absolute income for understanding anxiety related to consumptive behaviors^31^.

In Ukraine incomes were severely impacted due to a lack of employment opportunities from the shuttering and destruction of businesses and reduced consumption. Some statistics show a halving of Ukraine exports of agricultural commodities and metals and the destruction of 411 educational institutions, 36 healthcare facilities, 1600 residential buildings, 26 factories, and 6 thermal power plants/hydroelectric power plants^32^. Besides the loss of infrastructure, people’s direct experiences with violence have likely contributed to a sense of fear that restricts movements and reduces income and food access opportunities. For example, Rudolfsen et al.^2^ find that 60% of their respondents knew at least one person who had their person or property attacked in the two preceding weeks and that such experiences of violence are correlated with higher food insecurity.

Whereas direct destruction and violence in Ukraine helps explain reductions in income and mobility and high levels of food insecurity, the results in Switzerland point to economic, language and integration factors driving limitations in perceived income and food insecurity. Low employment is certainly one of the primary reasons for low income. A survey of the perceptions of Ukrainian refugees in Switzerland by the UN Refugee Agency found that only 21% of the refugees were employed in 2023, despite their high education level^33^. Within our sample we found that > 70% of the respondents had a higher education degree (Table S1), similar to the UN Refugee Agency survey. Yet the same UN survey found that language requirements remain a barrier to finding jobs as 77% of Ukrainian refugees report challenges with the local languages^34^.

Consequently, a vast majority of Ukrainian refugees in Switzerland, like other refugees to the country, rely heavily on public safety nets for income^35^. 43% of respondents to the UNHCR survey stating that aid from the government was their primary source of income^34^. These social security payments averaged around 1500 CHF / 1,572 USD per person per month depending on the Canton^35^, yet this is significantly lower than Swiss residents and other immigrant categories. The average annual income of foreigners with refugee and asylum permits in Switzerland 2022^36^ was CHF 55,212 (USD 57,868) per year, or CHF 4,601 (USD 4,822) per month, 40% lower than the annual income among Swiss nationals and 13% lower than other temporary permit holders.

Difficulties purchasing food in both places were likely exacerbated by inflation. The Swiss Consumer Price Index (CPI) increased by 2.8% in 2022^37^ while in Ukraine, it rose by 22.6% during the same period^38^. However, food prices in Switzerland were already extremely high compared to other countries^39^, explaining why even those with food aid may struggle to consume balanced meals, especially given their lower than socioeconomic status compared to Swiss nationals and permanent residences. Fresh foods, like fruit and vegetables, tend to be particularly expensive and out of reach for refugees in Switzerland^40^. Indeed, the UNHCR survey in 2023 found that 85% of refugees in Switzerland who responded to the survey were not receiving enough income to meet their basic needs (housing, food, basic commodities, school or medical expenses) in the past three months^34^. In Ukraine inflation was further exacerbated by a severe lack of mobility. Since the invasion and estimated 15,000 km of roads, 5,000 km of railways, 15 airports, and 350 bridges and overpasses were destroyed^32^.

The high level of food insecurity among both Ukraine residents and refugees to Switzerland underscores the dire challenges faced by those affected by conflict, especially poorer households, regardless of the wealth and conditions in the host country. While wealthier families can manage the situation better, poorer residents and refugees struggle to eat a balanced diet^41^. We find that families facing higher food insecurity more often utilized own food production, borrowing, and trading food to cope (Figure S1). Being able to produce one’s own food production was associated with lower food insecurity, but getting access to space to grow food is likely very difficult for refugees in Switzerland given the many year waiting lists for these spaces^41^.

Promisingly, lower income families in Switzerland were significantly more likely to receive food aid. Yet, women were less likely to have their own food production or be able to borrow or trade for food (Figure S1). We do not know the reason for these outcomes, but it may be due the fact that women more often have caretaking duties that restrict their time for other activities. For example, the UNHCR study found 88% of the single-headed households with dependents belonged to women^34^. The lower level of trading and borrowing for women may also be related to the higher level of judgement they already feel by Swiss society, as documented in a study of highly educated Syrian refugees in Switzerland^42^. In general, both male and female refugees face the serious challenge of not having a sufficient network to rely on in times of crisis with 20% of respondents to the UNHCR survey reporting have no one they can rely on in the event of emergencies^34^.

### Limitations

The conclusions that can be drawn from our analysis are limited by two major factors. First, the ability to reach refugees in Switzerland was limited by challenges partnering with the Secretary for Migration which restricted our ability to send the survey to Ukranian refugees in more targeted ways. The low resulting sample size restricted statistical inference. Second, we kept our survey short to avoid overburdening populations affected by the conflict. This reduced the number of explanatory factors we could examine in the regression. Nevertheless, our replicated survey in Ukraine and Switzerland has allowed us to uniquely provide a snapshot of the comparative food security outcomes of Ukranian residents versus refugees, painting a grim picture about effectiveness of efforts to curb food insecurity during the ongoing conflict.

## Conclusion

Our analysis focuses on the food security outcomes of Ukrainians who remained in the country after the Russian Invasion of 2022, as well as refugees to Switzerland, one of Europe’s wealthiest countries. These results provide only a small window into the negative effects of the Russian invasion of Ukraine on global food security. Disruptions to food, energy, and fertilizer production and distribution through port closures, the suspension of oilseed crushing operations and the introduction of export restrictions and bans for some crops and food products have also been noted in the literature. These factors have affected the incidence of severe food insecurity globally^43,44^ by pushing up food commodity prices, energy, and fertilizer prices^45–47^. The long-term legacy of these shocks on food systems, regional trade, and shifts in agricultural production throughout Europe and Eurasia are unknown, but it is likely that the impacts of conflict will leave a deleterious impact on global food security for years to come.

The Ukrainian population is one of the most well-educated on the planet and has received high levels of support from Europe during the conflict^48^. Ukranian refugees in Europe have benefited from special work opportunities^49^ and refugees to Switzerland have received significant aid from the Swiss government^34^. Despite this support, food insecurity is pervasive. It is likely that the food insecurity challenges are worse for refugees and households suffering from other conflicts globally.

Further study of the factors affecting the food insecurity of displaced peoples is urgently needed, especially since the number of refugees globally is expected to substantially increase in the future due to climate change^50^ and will be exacerbated by rollbacks in climate and development aid programs. The situation for Ukrainians remains particularly uncertain given massive changes in geopolitical relations in 2025, which will reduce support to populations living within Ukraine and likely catalyze further displacement.

## Supplementary Information

Supplementary Information.

## Data Availability

Aggregated data is provided within the supplementary information files (Tables S1-S4). Individual data cannot be shared.
