# Supplementary material for: The food security of residents and refugees of Ukraine after the Russian invasion
Source: Sci Rep. 2025 May 9;15:16238. doi: 10.1038/s41598-025-99285-1 (PMC12064787; doi:10.1038/s41598-025-99285-1)

Supplementary material for
"The food security of residents and refugees of Ukraine after the Russian invasion"

SM1 - Survey questions

FEED-UKRAINE: The politics of food security in Ukraine from the Russian invasion.
Food Security Survey

1. Country of residence
   [ ] Ukraine
   [ ] Switzerland

2. Canton in Switzerland or Region in Ukraine? __________________

3. If you are in Ukraine, do you live in the occupied territory?
   [ ] No
   [ ] Yes (Skip Survey)

4. What is your age group?
   [ ] 18-25 years
   [ ] 26-49 years
   [ ] 50-65 years
   [ ] Over 65 years

5. What is your nationality?__________________

6. How many people are in your household in Ukraine?__________________

7. If not currently in Ukraine, how many people are in your household in your current residence location___

8. How many people in the following age groups currently live in your household in your current residence location?
   [ ] ages 0–17_______# of household members
   [ ] 18–65__# of household members
   [ ] 65 +____# of household members

9. Which of the following best describes your gender identity?
   [ ] Female
   [ ] Male
   [ ] Non-binary

10. What is the highest level of formal education that you have?
   [ ] Some high school
   [ ] High school graduate
   [ ] Some college
   [ ] Associates degree/technical school/apprenticeship
   [ ] Bachelor's degree
   [ ] Postgraduate/professional degree

11. How do you estimate your income range in 2021?
   [ ] very low
   [ ] below average
   [ ] average
   [ ] above average
   [ ] high
   [ ] DK or Refused

[Begin food security module]

12. HH1: Which of these statements best *describes the food eaten in your household* since the Invasion of Ukraine, February 24, 2022?

[ ] Enough of the kinds of food I/we want to eat
[ ] Enough but not always the kinds of food I/we want
[ ] Sometimes not enough to eat
[ ] Often not enough to eat

13. HH2: *"(I/We) worried whether (my/our) food would run out before (I/we) got money to buy more."* Was this statement often true, sometimes true, or never true for (you/your household) since the Invasion of Ukraine, February 24, 2022?
[ ] Often true
[ ] Sometimes true
[ ] Never true

14. HH3: "*The food that (I/we) bought just didn't last, and (I/we) didn't have money to get more."* Was this statement often true, sometimes true, or never true for (you/your household) since the Invasion of Ukraine, February 24, 2022?
[ ] Often true
[ ] Sometimes true
[ ] Never true

15. *"(I/we) had access to sufficient, safe, and nutritious food that meets my dietary needs and preferences for an active and healthy life"* Was this statement often true, sometimes true, or never true for (you/your household) since the Invasion of Ukraine, February 24, 2022?
[ ] Often true
[ ] Sometimes true
[ ] Never true

16. HH4 *"(I/we) couldn't afford to eat balanced meals."* Was this statement often true, sometimes true, or never true for (you/your household) since the Invasion of Ukraine, February 24, 2022?
[ ] *Often* true
[ ] Sometimes true
[ ] Never true

17. Does your household receive food assistance (general in-kind food distribution and/or cash grants and/or food vouchers)?
[ ] Yes
[ ] No

18. How was the food eaten in your household acquired? (select all that apply)
[ ] Purchase
[ ] Own production (crops, livestock, fishing/ hunting, gathering) [ ] Traded goods/ services, barter
[ ] Borrowed (loan/ credit from traders)
[ ] Received as gift (from family relatives or friends/neighbour) [ ] In-kind or voucher/coupon-based food assistance
[ ] Other

Please answer the next 5 questions if you responded [3] or [4] to question 12 OR affirmative response (i.e., "often true" or "sometimes true") to 13, 14, OR 16

19. AD1: Since the Invasion of Ukraine, February 24, 2022, did (you/you or other adults in your household) ever *cut the size of your meals or skip meals* because there wasn't enough money for food?
[ ] Yes
[ ] No (Skip 19a)

19a. AD1a IF YES, how often did this happen?
[ ] Almost every month
[ ] Some months but not every month
[ ] Only 1 or 2 months

20. AD2 Since the Invasion of Ukraine, February 24, 2022, did you ever *eat less than you felt you should because there wasn't enough money* for food?
[ ] Yes

[ ] No

21. AD3 Since the Invasion of Ukraine, February 24, 2022, *were you ever hungry but didn't eat because there wasn't enough money* for food?
    [ ] Yes
    [ ] No

22. Since the Invasion of Ukraine, February 24, 2022, did you l*ose weight because there wasn't enough money* for food?
    [ ] Yes
    [ ] No

Please answer the next questions if you gave an affirmative response (yes) to any of the 5 questions above.

23. Since the Invasion of Ukraine, February 24, 2022, did (you/you or other adults in your household) ever *not eat for a whole day because there wasn't enough money* for food?
[ ] Yes
[ ] No

23a. IF YES, how often did this happen?
[ ] Almost every month
[ ] Some months but not every month
[ ] Only 1 or 2 months

Table S1: Mean food security scores by administrative unit 1

Urkaine Region	Mean Food Security Score	Swiss Canton	Mean Food Security Score
Kherson* Oblast	4.33	Schwyz	5.40
Mykolaiv* Oblast	4.14	Vaud	5.00
Kharkiv Oblast	3.62	Zug	5.00
Chernihiv* Oblast	3.48	Schaffhausen	5.00
Poltava Oblast	3.40	Solothurn	4.60
Donetsk* Oblast	3.00	Ticino	4.33
Rivne Oblast	2.97	Zurich	3.84
Vinnytsia Oblast	2.92	Basel Stadt & Land	3.00
Chernivtsi Oblast	2.90	Nidwalden & Obwalden	3.00
Odessa Oblast	2.82	St Gallen	3.00
Dnipropetrovsk Oblast	2.76	Valais	3.00
Zaporizhzhia Oblast	2.76	Neuchêtel	2.50
Sumy Oblast	2.73	Thurgau	2.50
Khmelnytskyi Oblast	2.70	Friburg	1.67
Kyiv	2.62	Geneva	1.08
Cherkasy Oblast	2.57	Glarus	1.00
Kyiv Oblast	2.54	Aargau	0.67
Volyn Oblast	2.39	Graubunden	0.50
Zhytomyr Oblast	2.34	Luzern	0.50
Lviv Oblast	2.09	Bern	0.43
Ternopil Oblast	2.03		
Kirovohrad Oblast	1.97		
Ivano-Frankivsk Oblast	1.97		

Notes: * indicates frontline territories.

Table S2. Background characteristics of respondents alongside broader country characteristics

Characteristic	Ukraine		Switzerland	
	Sample	Population	Sample	Population[e]
Median age	39	42[a]	41	41
Av. household size	3.3	2.6[b]	3.0	2.6
Women as % of population	79	60[c]	89	74, 90
Higher education	71	73[d]	79	70

[a]UN. 'Median Age'. UN Population Division Data Portal, 2025. https://population.un.org/dataportal/. [b]Ukrainian Center for Social Reforms. 'Ukraine - Survey of living conditions of households. State Statistical Committee of Ukraine, 2021 https://stat.gov.ua/. [c]UN Women. 'Rapid Gender Analysis of Ukraine'. United Nations Entity for Gender Equality and the Empowerment of Women; CARE International, 2022. https://www.unwomen.org/en/digital-library/publications/2022/05/rapid-gender-analysis-of-ukraine. [d]World Bank Data UNESCO Institute for Statistics 'School Enrolment – Tertiary (%)' 2021 https://data.worldbank.org/indicator/SE.TER.ENRR; [e]UNHCR. 'Switzerland - Survey of Intentions and Perspectives of Refugees from Ukraine'. Microdata Library, 2023. https://microdata.unhcr.org/index.php/catalog/1066

Table S3. Background characteristics of respondents alongside broader country characteristics

Characteristic	Category	Ukraine		Switzerland	
		Sample number	Sample %	number	%
Age	18-25 years	117	10.2%	5	4.7
	26-49	849	73.8	81	75.7
	50-65	181	15.7	18	16.8
	65+	4	0.3	3	2.8
	N/A	9	0.8	-	-
How many people are in the household	1	102	8.9	34	33.0
	2	278	24.3	19	18.4
	3	333	29.1	25	24.3
	4	234	20.4	9	8.7
	5	115	10.0	9	8.7
	6	42	3.7	2	1.9
	7	19	1.7	2	1.9
	8	11	1.0	1	1.0
	9	3	0.3	1	1.0
	10	4	0.3	1	1.0
	11	1	0.1	1	1.0
	12	2	0.2	1	1.0
	15	1	0	0	0
	Not answered	15	0.1	3	2.9
Gender	Female	790	68.5	94	89
	Male	356	30.8	10	9
	Non-binary	8	0.7	2	2
	Not answered	6	0.5	-	-
Education	Some high school	16	1	1	0.9
	High school graduate &/or some college	114	10	7	7
	Associates degree/technical school	204	18	14	13
	Bachelor's degree	153	13	11	10
	Postgraduate (advanced) degree	668	58	74	69

	Not answered	5	0.5	-	-
Perceived relative income range in 2021	Very low	143	12.4	14	13
	Below average	295	25.5	15	14
	- Total below average	438	37.9	29	27
	Average	574	49.7	52	49
	Above average	115	9.9	16	15
	High	11	1.0	4	4
	Not answered	22	1.9	5	5
Number of participants		1160		106	

Table S4

	**Model 1b - UA**	**Model 2b - UA**
Predictors	*Estimates (SE)*	*Estimates (CI)*
Perceived relative income group (1 = lowest, 5 = highest)	-0.57*** (0.24)	-0.57*** (0.07)
Advanced Education (1=Yes)	-0.27*** (0.07)	-0.24† (0.12)
Frontline (1=Yes)	1.00*** (0.12)	0.90† (0.47)
Food Aid (1=Yes)	0.25*** (0.14)	0.23 (0.15)
Own Production (1=Yes)	-0.58*** (0.13)	-0.59*** (0.13)
Household Size	0.10*** (0.04)	0.10*** (0.04)
Gender (1=Female)	0.04*** (0.13)	0.00 (0.13)
FE	No	Yes
Observations	1120	1120
R^2 / R^2 adjusted	0.12	0.13
Notes: Main models found in main text. Model 2b includes FE are at the region/canton level. †p<0.1, * p<0.05, ** p<0.01, *** p<0.001.		

Table S5: Switzerland regression results for the food insecurity index as the dependent variable

	**Model 1 - CH**	**Model 2 - CH**
Predictors	*Estimates (CI)*	*Estimates (CI)*
Perceived relative income group (1 = lowest, 5 = highest)	-0.48* (0.22)	-0.43† (0.24)
Advanced Education (1=Yes)	-0.38 (0.48)	0.30 (0.53)
Food Aid (1=Yes)	-0.05 (0.45)	-0.15 (0.53)
Own Production (1=Yes)	-0.01 (0.57)	0.04 (0.66)
Household Size	-0.01 (0.10)	0.06 (0.11)

Gender (1=Female)	-1.11<br>(0.71)	-0.78<br>(0.77)
Observations	1120	1120
Adjusted R^2	0.117	0.123

Table S6. Cantonal statistics

Canton	Average Food Security Score	Number of S Permits	Employment rate of S permit holders	GDP 2021 in CHF Millions	GDP per capita 2021 in CHF Millions
Schwyz	5.4	1,192	20.3%	10,807	66,333
Vaud	5	6,199	8.8%	62,917	76,834
Solothurn	5	2,116	15.5%	19,402	69,576
Zug	5	940	24.5%	22,570	174,570
Schaffhausen	5	674	14.5%	8,250	98,741
Ticino	4.33	2,683	9.2%	33,181	94,377
Zurich	3.84	11,363	17.9%	152,547	97,846
Basel Stadt & Land	3	4002	12.35%	30836	139,028
St Gallen	3	3,899	17.6%	41,979	81,218
Valais	3	2,358	9.6%	20,134	57,387
Nidwalden & Obwalden	3	579	24.1%	2938	71,625
Thurgau	2.5	1,952	24.1%	19,274	67,764
Neuchâtel	2.5	1,312	5.5%	17,603	99,999
Friburg	1.67	2,515	8.4%	20,252	61,808
Geneva	1.08	4,189	6.0%	56,342	110,932
Glarus	1	342	20.7%	2,980	72,650
Aargau	0.67	4,901	20.7%	44,951	64,346
Luzern	0.5	3,129	16.0%	30,128	72,019
Graubunden	0.5	1,451	21.5%	15,455	76,991
Bern	0.43	7,854	17.9%	84,878	81,199

Sources: Federal Statistical Office: Asylum statistics February 2023 https://www.sem.admin.ch/dam/sem/de/data/publiservice/statistik/asylstatistik/2023/02/6-24-Best-S-Erwerb-d-2023-02.xlsx.download.xlsx/6-24-Best-S-Erwerb-d-2023-02.xlsx & National accounts https://www.bfs.admin.ch/bfs/en/home/statistics/national-economy/national-accounts/gross-domestic-product-canton.html

Figure S1: Correlation matrices. Insignificant results (p>0.05) are blank.

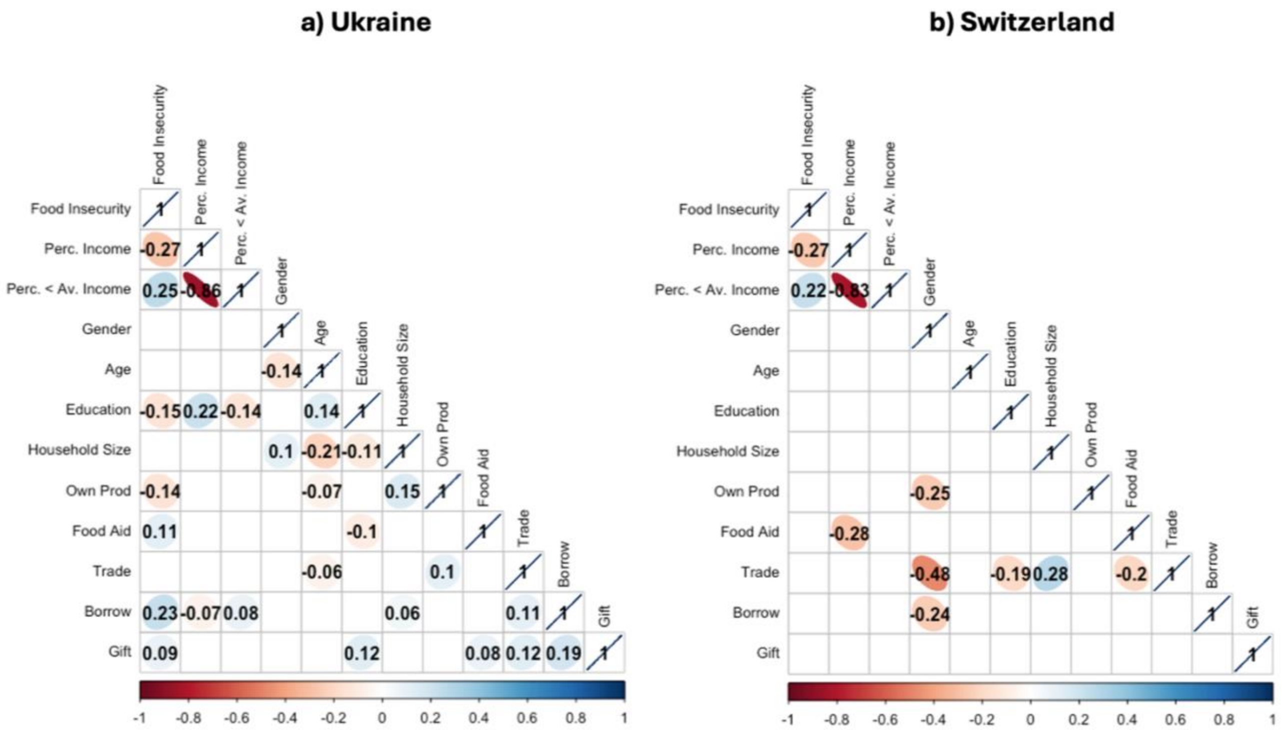

Supplement: Supplementary file 1 — Supplementary Information. [file 41598_2025_99285_MOESM1_ESM.pdf]
